# Integrating immunotherapy with conventional treatment regime for breast cancer patients- an amalgamation of armamentarium

**DOI:** 10.3389/fimmu.2024.1477980

**Published:** 2024-11-01

**Authors:** Deeptashree Nandi, Dipali Sharma

**Affiliations:** Department of Oncology, Johns Hopkins University School of Medicine and Sidney Kimmel Comprehensive Cancer Center at Johns Hopkins, Baltimore, MD, United States

**Keywords:** immunotherapy, breast cancer, triple-negative breast cancer, combination therapy, treatment

## Abstract

Immunotherapy stands as the frontrunner in treatment strategies imparting efficient remission in various types of cancer. In fact, emerging breakthroughs with immune checkpoint inhibitors (ICI) in a spectrum of cancers have evoked interest in research related to the potential effects of immunotherapy in breast cancer patients. A major challenge with breast cancer is the molecular heterogeneity that limits the efficacy of many therapeutic regimes. Clinical trials have shown favorable clinical outcomes with immunotherapeutic options in some subtypes of breast cancer. However, ICI monotherapy may not be sufficient for all breast cancer patients, emphasizing the need for combinatorial approaches. Ongoing research is focused on untangling the interplay of ICI with established as well as novel anticancer therapeutic regimens in preclinical models of breast cancer. Our review will analyze the existing research regarding the mechanisms and clinical impact of immunotherapy for the treatment of breast cancer. We shall evaluate the role of immune cell modulation for improved therapeutic response in breast cancer patients. This review will provide collated evidences about the current clinical trials that are testing out the implications of immunotherapy in conjunction with traditional treatment modalities in breast cancer and summarize the potential future research directions in the field. In addition, we shall underline the recent findings related to microbiota modulation as a key regulator of immune therapy response in cancer patients and its plausible applications in breast cancer.

## 1 Introduction

Breast cancer persists as a global health menace, accounting for one-third of all new cancer cases and ranking as the second most prevalent malignancy among women (1). With more than 2.3 million cases worldwide, breast cancer incidence is currently on the rise. Depending on the type of hormone receptors being expressed on the breast carcinoma cells, there exist four primary molecular subtypes of breast cancer; estrogen receptor/progesterone receptor (ER/PR)-positive but HER2-negative (luminal A) that comprises of more than half of the breast cancer cases; ER/HER2-positive but PR-negative (luminal B)- hormone therapy as well as chemotherapy may be effective for treating both luminal A and luminal B subtypes; ER/PR-negative but HER2-positive (HER2 positive)- this group of tumors are likely to respond to HER2-targeted therapy; and triple-negative breast cancer (TNBC, basal-like) with too little to no expression of any of the receptors, making it the most challenging breast cancer subtype to target (2). Importantly, TNBC makes up about 10-15% of all breast cancers and is the most aggressive form of this malignancy. Given the complex heterogeneity with diverse molecular subtypes and various underlying genetic alterations, the choice of treatment and the therapeutic response varies greatly among breast cancer patients. At present, surgical resection, chemotherapy, and radiotherapy are the frontline treatment approaches for managing locally advanced breast cancer. Endocrine therapies such as SERM tamoxifen, SERD fulvestrant, or the aromatase inhibitors anastrozole and exemestane, are well-accepted forms of targeted therapy for ER-positive breast cancer (3–5). Small molecule inhibitors against CDK4/6 (palbociclib, abemaciclib, ribociclib), PARP (olaparib, talazoparib, rucaparib, niraparib), PI3K/AKT, mTOR, FGF receptors and VEGF also hold strong potential as precision medicines to mitigate breast cancer progression due to their intimate involvement in oncogenic signaling pathways (6, 7). *De novo* and acquired resistance to several therapies has also been noted in breast cancer leading to the development of newer regimens that may prove effective against resistant tumors. For e.g., everolimus (42-O-(2-hydroxyethyl) rapamycin), an mTOR inhibitor, is approved for advanced or metastatic ER-positive breast cancer that no longer responds to aromatase antagonist. Despite their clinical prowess, therapeutic resistance severely limits the efficacy of several drugs.

The emergence of immunotherapy as the fourth pillar of anticancer strategies has helped prolong the survival of several breast cancer patients. Immune checkpoint inhibitors (ICIs) encompassing CTLA-4, PD-1, and PD-L1 inhibitors have been authorized for treating solid tumors, including breast cancer. Multiple clinical trials, conducted over the years, led to the FDA approval of the first ICI-ipilimumab, a CTLA-4-blocking antibody in 2011 for metastatic melanoma (8). The ensuing investigations resulted in the development of several PD-1-targeting antibodies that were markedly effective in clinical settings, leading to the subsequent approval of nivolumab and pembrolizumab in 2014. These successes fueled further research endeavors helping the development of inhibitory antibodies against additional targets such as PDL1, LAG3 protein, hepatitis A virus cellular receptor 2 (also known as TIM3), and T cell immunoreceptor with Ig and ITIM domains (9). Moreover, efforts have been directed to engage T cell immune response *via* agonist antibodies that function primarily by activating receptors on the immune cells, like CD40, and TNF receptor superfamily member 9 and 4 (10). Immunotherapy repertoire has shown very promising responses in multiple cancer types while progress in breast cancer has been rather slow. Contrary to the older notion of a “poorly immunogenic” nature, current research strongly indicates that breast tumors are composed of a complex, heterogenous and dynamic network of untransformed epithelial cells, genetically modified cancer cells, fibroblasts, immune cells, and blood vessels. There exists an intricate communication among these different constituents. Also, these components interact with the surrounding microenvironment which changes with cancer progression and in response to therapy. Improved understanding of the dynamic breast cancer microenvironment has led to tremendous progress in the development of immunotherapy in breast cancer.

## 2 Current status of immunotherapy for different subtypes of breast cancer

Multiple investigations have improved our current knowledge of immune evasion by tumor cells and enabled the development of specific immune checkpoint inhibitors as state-of-the-art therapeutic choice. Immunotherapy primarily entails boosting the host immune system so as to enable it to recognize cancer cells as a foreign invader and subsequently destroying them. The previously believed notion about the ‘non-immunogenic environment of breast cancer’ has been challenged with the discovery of tumor-infiltrating lymphocytes (TILs) in breast tumors (11). Of note, HER2-positive and TNBC subtypes demonstrate an elevated number of TILs compared to the other breast cancer subtypes (12, 13). Currently, there is an increasing interest in the application of immune checkpoint blockers to treat breast cancer patients who are refractory to any other forms of treatment.

### 2.1 Immunotherapeutic approaches for the treatment of HER2-positive breast carcinoma

Clinical trials in patients with HER2-positive breast cancer have exhibited modest results with immunotherapy. The combination of anti-PD-1 mAb (pembrolizumab) and trastuzumab was assessed for the treatment of HER2-positive progressive metastatic breast cancer, wherein a partial response was achieved in 15% of the enrolled patients with PD-L1 positive tumors (14). Moreover, dendritic cell (DC) vaccines that were primed against the HER2 protein proved beneficial in mammary tumor regression through activation of anti-HER2 CD4^+^ Th1 response in an early phase clinical trial (15). Preclinical investigations in immunocompetent mice suggest that PD-1 and CTLA-4 inhibition leads to a considerable increase in the immune-associated effects of HER2-based targeted therapies which is accomplished *via* synergistic activation of CD8^+^ T cells (16, 17). The PANACEA trial revealed that 15% of trastuzumab-resistant HER2-positive breast cancer patients harboring PD-L1-positive tumors elicited a partial response when treated with pembrolizumab plus trastuzumab (14). In the “Proceedings of the 2018 San Antonio Breast Cancer Symposium”, Emens et al. discussed the randomized phase II KATE2 trial, which revealed that patients with PD-L1-positive, HER2-positive, pre-treated metastatic breast cancer exhibited improved PFS following treatment with T-DM1 combined with atezolizumab relative to T-DM1 alone. Of note, CAR T-cell therapy, a popular example of adoptive T cell therapy, have proven successful in patients with hematological cancers and is currently being explored in solid tumors. Researchers have successfully expanded T cells specific for HER2 *ex vivo* in mice models and these were found to elicit antitumor activity (18). Administration of HER2 CAR T cells with CD28 costimulatory domain in the mice central nervous system resulted in the regression of HER2-positive metastatic breast carcinoma in the CNS (19). Nonetheless, clinical data regarding the application of adoptive T cell therapy for treating HER2-positive breast malignancy are still lacking. Preclinical and clinical observations, though limited, solidify the rationale for the clinical development of ICI for the treatment of HER2-positive breast carcinoma, and emphasize the need for more detailed research into the development of immunotherapeutic modules, especially in combination with HER2-targeted therapies.

### 2.2 Immunotherapeutic approaches for the treatment of triple-negative breast cancer

Reportedly, factors such as a heavier tumor mutation load, increased frequency of TILs and enhanced expression of PD-L1 may contribute to increased immunogenicity for TNBC, thus, TNBC patients are expected to benefit from ICIs (20). The efficacy of pembrolizumab monotherapy as a first-line of therapy for metastatic TNBC (mTNBC) patients was evaluated in the phase II KEYNOTE-086 study (21). The results from the study showed favorable anti-tumor activities with median PFS of 2.1 months whereas the median OS was improved to 18 months. Many investigations are additionally aimed at establishing pembrolizumab monotherapy as a second-line or later therapeutic strategy in pre-treated mTNBC patients, including the KEYNOTE-086 (21), the KEYNOTE-012 (22) and the TAPUR basket study (23). In the phase III KEYNOTE-119 trial, efficacy of pembrolizumab monotherapy was assessed in comparison to standard chemotherapy in second or third line of treatment for patients with mTNBC. However, there was no evident improvement on the prognosis of 622 TNBC patients, who had progressed on 1-2 cycles of either taxane or anthracycline (24). Such results indicate the immediate need for additional large-scale randomized controlled trials and the need for combination approaches, especially in earlier lines of treatment. Atezolizumab and durvalumab are two more anti-PD-L1 antibodies that are yielding promising results (22). A phase II trial, consisting of 199 patients with no disease progression after 6-8 cycles of chemotherapy, has evaluated the role of durvalumab for TNBC treatment. In the exploratory subgroup analysis of TNBC patients (n = 82), durvalumab dramatically increased the OS (25), suggesting the rationale for additional investigations into using durvalumab as a therapy for TNBC patients with advanced disease. Avelumab, another PD-L1 inhibitor, is currently being tested as second-line or posterior-line therapy at JAVELIN basket trial, which has shown some promising results (26). Findings from the phase II TONIC study have indicated that addition of cisplatin and doxorubicin may exert better tumor response to immunotherapy in TNBC patients (27). The detailed insight into the underlying molecular mechanisms is still not thoroughly understood and remains an imperative area of future research focus. Nonetheless these reports strongly support the improved and durable clinical efficacy of PD-1/PD-L1 inhibition as an effective treatment modality for patients with TNBC.

### 2.3 Attempts evaluating immunotherapeutic approaches for the treatment of luminal A/B breast cancer

It is important to note that not all subtypes of breast carcinoma equally respond to the effects of immunotherapy. For instance, in subjects bearing the luminal subtype of breast cancer, initial attempts used a combination of ICI with chemotherapy as a novel form of anti-cancer therapy- but that yielded disappointing results. One such study aimed at investigating the tumor suppressive effects of pembrolizumab combined with eribulin among patients harboring ER/PR-positive, HER2-negative metastatic breast carcinoma (28). However, the combination therapy did not lead to any noticeable improvement in the clinical outcome or prognosis of the metastatic luminal A-subtype breast cancer patients- the most possible reason underlying this pertains to the heavily-pretreated nature of the subjects in the study. A phase Ib non-randomized, open-label, multi-cohort study tried to evaluate the clinical impact of pembrolizumab plus abemaciclib in the presence or absence of anastrozole (endocrine therapy) in metastatic breast cancer patients with ER-positive, HER2-negative subtype. Patients had no prior exposure to CDK4/6 inhibitor treatment (29). Unfortunately, the anti-tumor efficacy of the combination was mitigated by the appearance of toxicity in the liver and lungs following therapy. In contrast, administration of letrozole, palbociclib, and pembrolizumab as front-line therapy in HR-positive, HER2-negative metastatic breast carcinoma was associated with good tolerance and favorable clinical efficacy in a phase I/II trial (30). For luminal B- subtype, the neoadjuvant phase II GIADA trial subjected patients to sequential anthracycline-associated chemotherapy prior to the treatment with nivolumab and endocrine therapy (31). A 16.3% pCR rate was observed followed by the identification and characterization of immune-based gene signatures and immune sub-populations that were correlated with the achieved pCR. While previous studies have found no notable benefit from pembrolizumab in a metastatic setting of the luminal subtype, addition of pembrolizumab to a sequential cycle of chemotherapy in a neo-adjuvant setting was found to elevate the pCR rate from 13 to up to 30% amongst patients with luminal breast cancer (32).

## 3 Development of novel treatment modalities combining immunotherapy with existing and upcoming therapeutic agents to target breast cancer

**Figure 1:**
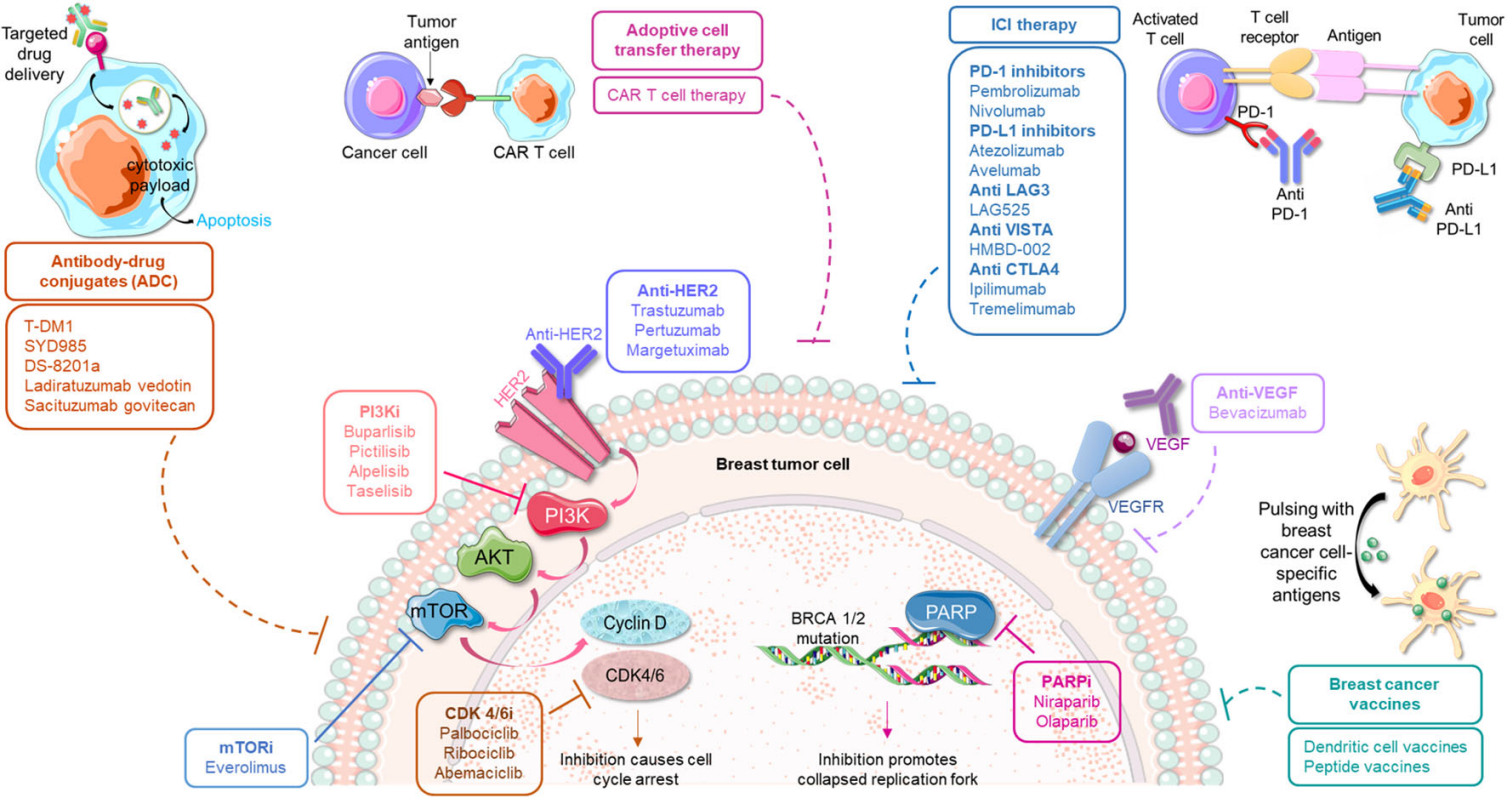
Immunotherapeutic strategies in conjunction with traditional modes of anticancer treatment approaches for the management of breast cancer. The predominant small molecule inhibitors used for treating breast cancer include inhibitors against PI3K, mTOR, CDK 4/6 and PARP. These agents can be potentially used in combination with various emerging immune-therapies, such as, anti-PD-1/anti-PD-L1 therapy, antibodies against CTLA-4 and other immune checkpoints, different antibody-drug conjugates, adoptive cell transfer therapy as well as specific vaccines. Such combinatorial regimes are currently being investigated for their safety and efficacy in breast cancer.

Contemporary research is focused on exploring the synergistic effects of ICIs and commonly used chemotherapies for treating breast carcinoma patients as monotherapy approaches using ICIs exhibited modest activity. Chemotherapy is well-known to repress the actions of immunosuppressive cells, like MDSCs and T_reg_ cells, while simultaneously facilitating cancer cell apoptosis, promoting tumor antigen cross-presentation, and exacerbating recruitment and infiltration of CD8^+^ T cells, NK cells and DCs *via* the secretion of pro-inflammatory cytokines in the macrophages. Preclinical evidences from animal models and clinical studies are already recognizing the intricate drug-dependent and dose-dependent interplay between chemotherapy and the immune system- thus, this interaction can be exploited for synergistic associations between cytotoxic drugs and immunotherapy (**Figure 1**).

### 3.1 Combination modalities involving immunotherapy and HER2-targeted therapies

Utomilumab is a receptor IgG2 mAb agonist against 4-1BB, a co-stimulatory receptor that is involved in immune cell proliferation once activated. For the treatment of patients with advanced HER2-positive breast cancer, a phase I dose-escalation trial is investigating the combination of utomilumab with either trastuzumab or T-DM1 (NCT03364348). The effects of utomilumab plus avelumab is also being studied in a phase II trial (AVIATOR, NCT03414658). The preclinical findings revealed that utomilumab, when combined with a mAb targeting the PD-1/PD-L1 axis, can aid in a strong immune response (33). Another component associated with the adaptive immune response is the toll-like receptor, and activation of TLR4 can stimulate antigen processing and cross-presentation *in vivo (* 34). In pre-clinical models, oligodeoxynucleotides with CpG motifs that activate TLR9 have been shown to induce active immune cytotoxicity (35). Activation of TLR2 in HER2-positive breast cancer preclinical models augments trastuzumab-related cytotoxicity (36). Such results inspired research undertakings for testing the therapeutic efficacy of TLR agonists in combination with HER2-based vaccines (NCT02276300). Synergistic interactions between trastuzumab and docetaxel chemotherapy have yielded a 60% response rate compared to 11% in the case of monotherapy (37). Taking these observations forward, clinical trials inspecting the efficacy of combining atezolizumab with HER2 mAbs plus chemotherapy in patients who are receiving early first line therapy for HER2-positive breast cancer are in progress. The findings from such trials are likely to provide new avenues for the treatment modalities (NCT03125928, NCT03726879) (38). While approaches combining immunotherapy and HER2-targeted therapies are promising candidates for the treatment of HER2-positive breast cancer, they can also be useful for a wider patient population including tumors exhibiting a modest/low HER2 expression level which are not usually eligible for HER2 mAbs.

### 3.2 Integration of immunotherapy with chemotherapy regimens for improved outcomes

One-third of patients with TNBC experience distant recurrences, and eventually succumb to death within 5 years post- diagnosis. Therefore, TNBC has a dire need for superior treatment options and precision medicine. A phase III clinical study, IMpassion130, interrogated the impact of immunotherapy in 902 patients with advanced TNBC, and tested the efficacy and safety of the PD-L1 antibody atezolizumab in conjunction with chemotherapeutic drug nab-paclitaxel (39). The promising data showed a significant improvement in the mean OS from 15.5 to 25 months among PD-L1-positive patients (40). The findings led to the FDA approval of the combination of atezolizumab and nab-paclitaxel for therapy-naïve patients having PD-L1-positive advanced TNBC in 2019 (41). Follow up phase III trials IMpassion131 and Impassion 132 are delineating the clinical impacts of atezolizumab with paclitaxel or first-line chemotherapy (carboplatin, gemcitabine or capecitabine) in multiple settings of TNBC (42). The primary goal of the IMpassion131 trial was to test the efficacy of weekly administration of paclitaxel as the chemotherapy backbone plus atezolizumab in a group of patients constituting of similar inclusion criteria as IMpassion130. Unfortunately, the results from this trial were not in sync with the observations from Impassion130. Of note, in an abstract presented at the “2021 ASCO Annual Meeting”, it was shown that several differences were present in the tumor microenvironment of tumor samples from patients enrolled in the two trials. Additional reasons underlying the observed discrepancy in results may be attributed to the differences in the body mass index and gut microbiota composition among the enrolled patients (43). Another phase III trial, KEYNOTE-355, tested the effects of integrating pembrolizumab with chemotherapy (albumin-bound paclitaxel, paclitaxel or gemcitabine/carboplatin) for the treatment of locally recurrent, inoperable or mTNBC patients who have not undergone prior therapy. There was a considerable prolonged PFS among PD-L1 positive population in the pembrolizumab-based group (44), paving the way for the accelerated FDA approval of pembrolizumab in combination with chemotherapy for the treatment of patients with locally recurrent unresectable or metastatic PD-L1-positive TNBC. Final results of the KEYNOTE-355 trial reported that the combination considerably benefitted the patients with a mean OS of 23 and 16.1 months in the combination versus chemotherapy alone group, respectively. Intriguingly, 22% of the TNBC patients in the pembrolizumab arm boasted of a disease-free interval between 6 and 12 months. Results from a phase II randomized trial demonstrated that chemotherapy or radiotherapy promoted a more favorable tumor microenvironment in TNBC patients that boosted the response to PD-1 blockade through nivolumab. Patients subjected to the combination regimen that included immunotherapy experienced a clear improvement in their median DFS and OS, relative to the individuals treated with monotherapy alone (27). Of interest, Oleclumab, a mAb specific for CD73, is being studied in the phase Ib/II BEGONIA study, as a combination therapy with durvalumab, plus paclitaxel, as first-line treatment for mTNBC individuals (NCT03742102). In the ENHANCE 1 trial enrolling 104 patients with mTNBC, eribulin, a microtubule inhibitor, when administered with pembrolizumab, displayed attractive tumor-suppressive activity (45). An objective response was achieved in (i) 26% of the evaluable patients, (ii) 25% of the 48 patients who were not exposed to any prior chemotherapy and (iii) 27% of the 34 subjects who had previously received chemotherapy. Again, in agreement with observations from other trials, patients harboring PD-L1-positive breast cancer boasted a better response than those having PD-L1-negative tumors. The exciting findings from such trials have elicited investigations into various combination regimes among TNBC patients. Chemotherapy regimens continue to be the frontline treatment strategy for a majority of breast cancer patients; however, it is associated with adverse side effects affecting the quality of life as well as therapy resistance leading to suboptimal response. While immunotherapy regimens are still being investigated for their long-term impact on quality of life, the combination treatment strategies combining chemotherapy and immunotherapy are presenting improved responses than monotherapy alone.

### 3.3 Development of novel combinations using immunotherapy with antiangiogenic agents, HDAC inhibitors and topoisomerase inhibitors

Multiple studies are underway focusing on determining the efficacy of immunotherapy with anti-angiogenesis agents. The GINECO A-TaXel phase II trial in TNBC reported a significant activity and tolerable safety profile for the combination of paclitaxel, capecitabine and bevacizumab, a recombinant humanized mAb against VEGF-A (46). In 2019, a single-arm trial investigated the effects of Nivolumab, paclitaxel and bevacizumab as first-line therapy in patients with HER2-negative metastatic breast carcinoma, consisting of both hormone-positive and TNBC populations. The treatment group exhibited a PFS of 8.1 months among the TNBC individuals and 19.1 months in the hormone receptor-positive subgroup (47). In addition, in heavily pretreated patients with advanced TNBC, a novel humanized mAb targeting PD-L1, TQB2450, was tested in combination with anlotinib, an anti-angiogenic small molecule inhibitor- the combination arm displayed an acceptable safety profile with potent activity (48). The capacity of HDAC inhibitors in upregulating antigen presentation genes and boosting tumor cell recognition by activated ICs suggest that they may act in augmenting the efficacy of ICIs. The cocktail of romidepsin, cisplatin and nivolumab indicated encouraging signs of efficacy in 34 pre-treated mTNBC patients, necessitating additional research in larger populations (NCT02393794). In contrast, evidences from a phase II study in people with advanced TNBC suggest that another HDAC inhibitor, entinostat, failed to improve PFS in combination with atezolizumab (NCT02708680), supporting the dire need for further investigation of the combination. With advances in research related to ADC, a randomized phase II trial is affirming the impact of pembrolizumab plus sacituzumab govitecan, composed of a topoisomerase I inhibitor (SN-38) and an anti-Trop2 monoclonal antibody, in patients with PD-L1 negative mTNBC (NCT04468061). Overexpression of Trop-2 is predictive of a more aggressive TNBC (49). Sacituzumab govitecan was found to serve as a potent immunomodulator, promoting antibody-driven cytotoxicity, depletion of T_reg_ cells and upregulation of MHC class I and PD-L1 expression in mice models, and it may overcome resistance to current immunotherapeutic strategies in PD-L1-negative tumors, which forms the rationale of the clinical trial. A recent presentation at the “2020 AACR annual meeting” demonstrated the potential medical application of another ADC with similar immunomodulatory features, ladiratuzumab-vedotin, an anti-LIV-1 ADC, in conjunction with pembrolizumab as first-line therapy in TNBC patients in a phase Ib/II study- the combination proved tolerable and exhibited promising anticancer activity. ICI therapy impedes the tumor-promoting signals that enable immune evasion by cancer cells. Combining this method with agents that function by potently targeting various other hallmarks of cancer, such as angiogenesis, epigenetics modulation and DNA damage response, can potentially results in synergistic effects that will ultimately lead to more successful response in breast carcinoma patients.

### 3.4 Examining combinations of immunotherapy with multiple kinase inhibitors

Various kinase inhibitors have been tested to target specific oncogenic pathways in breast cancer. Currently, many clinical studies are exploring combination regimens involving kinase inhibitors and immunotherapy. The phase II COLET study evaluated the therapeutic efficacy of atezolizumab, MEK1/2 inhibitor cobimetinib, and nab-paclitaxel or paclitaxel in locally advanced or mTNBC, wherein PD-L1-positive patients accomplished a visibly higher tumor ORR and PFS than PD-L1-negative individuals (50). The therapeutic outcome of combining PD-1 monoclonal antibody camrelizumab with apatinib for treating advanced TNBC was tested in a phase II study (51). The results were intriguing as they revealed an ORR as high as 32.5% compared to 18.5%, which is the highest recorded ORR for anti-PD-L1 monotherapy in TNBC, paving the groundwork for an effective alternative combinational approach for TNBC treatment. Moreover, a prospective phase II trial (FUTURE-C-PLUS) is ongoing that seeks to assess the efficacy and safety index of the combination of camrelizumab plus chemotherapy (nab-paclitaxel) and famitinib (multityrosine kinase inhibitor against VEGFR-2, PDGFR and c-kit) in mTNBCs. A major part (81.3%) of the population achieved objective responses with a 60.2% of 9-month PFS rate (52). These promising results inspired the ongoing phase II randomized trial FUTURE-SUPER (NCT04395989). A phase Ib/II study is presently determining the effects of tislelizumab, an anti-PD-1 IgG4-variant mAb, in combination with fruquintinib, a highly selective, oral tyrosine kinase inhibitor of VEGFR, in mTNBC, including patients who have been pretreated with immunotherapy in addition to naïve patients (NCT04579757). Meanwhile, patients suffering from AR-positive metastatic TNBC, when subjected to pembrolizumab combined with the AR regulator GTx-024 in a phase II clinical trial, demonstrated an ORR of 25% at 16 weeks (53). In another study, an AKT inhibitor ipatasertib was subsequently combined with the atezolizumab and paclitaxel or nab-paclitaxel cocktail as another candidate for front-line treatment. Irrespective of the expression of PD-L1 or alteration status of PIK3CA/AKT1/PTEN, 19 out of 26 patients showed a response accompanied with a significantly elevated ORR of 73%, thus advocating a novel therapeutic regime for treating TNBC (54). In addition to their traditional role in targeted inhibition of key proteins involved in cell survival and growth, kinase inhibitors eradicate tumors *via* certain immune-modulatory effects. Immunotherapy, when used in conjunction with such precision therapy, can suppress the toxicities associated with monotherapy and impart improved targeted anti-tumor functions even in breast cancer patients, who do not respond well to immunotherapy alone.

### 3.5 Combining PARP inhibitors and CDK inhibitors with immunotherapy as a new arsenal for targeting breast cancer

A more recent undertaking, which is currently recruiting patients with locally advanced or metastatic HER2-negative mammary carcinoma with homologous DNA repair deficiency, aims to uncover the efficacy of atezolizumab when incorporated with the PARP inhibitor, olaparib (NCT02849496). The TOPACIO/KEYNOTE-162 trial is a single-arm phase II study in advanced TNBC population that found a considerable anti-tumor activity and manageable safety profile for the combination of pembrolizumab and a PARP inhibitor, niraparib (55). Importantly, this combination was especially beneficial for patients harboring tumors with BRCA mutation. Furthermore, niraparib synergistically potentiates the anticancer functions of the anti-PD-1 antibody, BioXCell RMP1-14, in TNBC models through activation of the interferon signaling (56). Furthermore, co-administration of anti-PD-L1 monoclonal antibody, durvalumab and olaparib in advanced breast carcinoma with genomic BRCA mutation exhibited better survival rates in a phase I/II MEDIOLA study (57), denoting the alluring prospect of integrating PARP inhibitors with immunosuppressants as an efficient anticancer module for TNBC patients. Moreover, the SGNLVA-002 study attempts to assess the effects of the novel combination of pembrolizumab with ladiratuzumab vedotin, an ADC with great potential, as a front-line treatment choice for locally advanced or mTNBC (NCT03310957). A phase II randomized controlled trial in 2019 unveiled that PD-L1-positive or TNBC patients demonstrated a pronounced benefit when subjected to treatment with durvalumab with the median OS of durvalumab-treated PD-L1 positive or TNBC patients being 21 months and 26 months, respectively (NCT02299999). The efficacy of durvalumab plus the PARP inhibitor, olaparib, as a maintenance strategy for patients with platinum-sensitive advanced TNBC is being determined in the DORA study (NCT03167619). Another category of agents that holds imminent interest in combination modules with ICIs are the CDK inhibitors. In preclinical models of TNBC, dinaciclib, an intravenous CDK inhibitor, potentiated the antitumor effects of ICI through heightened immune cell activation and tumor infiltration. Following this, a phase Ib, dose-escalation trial tested dinaciclib plus pembrolizumab in patients with advanced TNBC, which revealed manageable toxicities upon reduction and delayed administration of the specified dose while the dose expansion part is ongoing. Furthermore, the CDK4-6 inhibitor, palbociclib, is being interrogated in combination with avelumab in AR-positive TNBC (NCT04360941). DNA-damaging agents, such as PARP inhibitors, can potentiate immune response through enhanced tumor mutational burden and improved neoantigen release, thereby rendering the tumor more amenable to immunotherapy. CDK4/6 inhibition is known to impart transcriptional reprogramming of immune as well as tumor cells, resulting in higher immunogenicity of cancer cells and an immune-rich TME, which is, again, more susceptible to immune-based therapies. Therefore, combining these approaches with immunotherapy can lead to positive response rates in a number of breast cancer patients, including those who are originally less responsive to ICI therapy.

### 3.6 Combining multiple immunotherapy regimens to enhance the clinical efficacy

Recent studies are also investigating the utility of combining different ICI regimes. Multiple CTLA-4 inhibitors have shown the efficacy in combination therapy for solid tumors, including breast carcinoma. While ipilimumab was FDA approved for better survival among advanced metastatic melanoma patients, its anti-tumor effect was modest in TNBC (58). A single-arm clinical study in patients with metastatic breast cancer, including TNBC population, tested the efficacy of durvalumab in conjunction with tremelimumab but the trial did not meet a successful completion (59).Treatment with dual anti-PD-1 and anti-CTLA-4 plus cisplatin resulted in activation of DCs and CD8^+^CD4^+^ T cells along with suppression of FOXP3^+^ T_reg_ cells and the effect was more pronounced in BRCA-1 deficient tumors (60). LAG3 is an immune checkpoint that blocks the activation of its host cell to facilitate further suppression in the immune response. LAG525 is an antibody raised against LAG3, which was tested in the setting of mTNBC in a phase II trial, in conjunction with PDR001, an anti-PD1 antibody in the presence or absence of carboplatin (61). The triplet arm showed an ORR of 32.4%. ICOS is a member of the CD28 superfamily that interacts with ligands expressed on B cells and phagocytes, thus promoting downstream signaling to regulate T cell proliferation and release of cytokines. In a phase I/II open-label study involving patients with advanced solid tumors, KY1044, a human anti-ICOS antibody, was tested as monotherapy and in combination with atezolizumab. KY1044 was well-tolerated in both the strategies and one complete response and four partial responses were noted in the TNBC cohort (NCT03829501). Results from the phase II part of the study are underway. Another immune-regulatory protein that suppresses T cell activation and cytokine production, VISTA, is capable of inducing an immunosuppressive environment. HMBD-002 is an antibody against VISTA, which is currently being studied in a phase I study among patients with advanced TNBC (NCT05082610). A summary of the clinical studies involving a combinatorial approach of immunotherapy plus some form of traditional anticancer therapeutic module that have demonstrated safe and favorable disease outcome so far is presented in **Table 1**.

**Table 1:** Clinical trials in breast cancer subjects pertaining to the combination of immunotherapy with different forms of conventional anticancer treatment strategies.

Combined therapy	Anti-PD-1/PD-L1	Another agent	Phase	Number of patients	Conclusions	Disease setting	Clinical Trial number
**HER-2-targeted**	Pembrolizumab	Trastuzumab	I/II	58	Safe; with activity and durable clinical benefit in PD-L1/HER2, trastuzumab-resistant, advanced breast cancer patients; disease control achieved in 25% of patients in the PD-L1-positive subgroup.	Trastuzumab-resistant, advanced, HER2-positive breast cancer	NCT02129556
	Durvalumab	Trastuzumab	I	15	No objective response; best outcome was stable disease at week 6 in 4 (29%) of 14 patients with PD-L1-negative cancers.	HER-2 positive metastatic breast cancer progressing on prior anti HER-2 therapies	NCT02649686
**PARPi**	Pembrolizumab	Niraparib	I/II	55	Safe, promising antitumor activity; objective response was achieved by 13 (29%) of 45 evaluable patients overall, 8 (67%) of 12 patients with genomic BRCA mutations, 33% of patients with PD-L1-positive cancers and 15% of patients with PD-L1-negative cancers.	Advanced or metastatic TNBC	NCT02657889
	Durvalumab	Olaparib	I/II	288	Objective response was achieved by 67% of patients in the first-line setting group.	Germline BRCA-mutated, metastatic breast cancer	NCT02734004
**Chemotherapy**	Atezolizumab	Nabpaclitaxel	Ib	33	Safe, nab-paclitaxel did not impair systemic immune activation by atezolizumab; objective response from 13 (39%) patients overall and from 7 (54%) of 13 who received atezolizumab as first-line treatment.	Metastatic or locally advanced TNBC	NCT01633970
	Durvalumab	Anthracycline/taxane	II	117	Increases pCR rate in patients treated with durvalumab alone prior to start of chemotherapy; better response in PD-L1 tumors.	Early TNBC	NCT02685059
	Atezolizumab	Nabpaclitaxel	III	902	Median PFS was 7.2 months (95%) and median overall survival was 21.3 months (95%) for all patients; median overall survival in the PD-L1-positive subgroup was 25.0 months (95%).	Previously untreated, locally advanced or metastatic TNBC	NCT02425891
	Pembrolizumab	Chemotherapy	III	1372	Significantly longer overall survival than chemotherapy alone in patients with advanced TNBC with PD-L1-positive tumors.	Previously untreated, locally recurrent, inoperable or metastatic TNBC	NCT02819518
**Tyrosine-kinase inhibitor**	Camrelizumab	Apatinib	II	40	Median PFS of 8.1 (95%) months; grade 3/4 treatment-related adverse events occurred in 19 (41.3%) of 46 patients; combination showed promising efficacy with a measurable safety profile in patients with heavily pretreated advanced TNBC.	Metastatic or unresectable recurrent TNBC	NCT04303741
**Cyclin-dependent kinase inhibitors**	Pembrolizumab	Abemaciclib	Ib	28	Objective response was achieved in 14% of patients with HR-positive, HER-negative metastatic breast cancer.	HR-positive HER-negative breast cancer	NCT02779751
**Microtubule inhibitor**	Pembrolizumab	Eribulin	Ib/II	167	The combination was generally well tolerated and showed encouraging antitumor activity with an ORR of 23.4%, median OS of 15.5 months and median PFS of 4.1 months.	Metastatic TNBC	
**Anti-PD-1 and ** **anti-PD-L1 combinations**	Durvalumab	Tremelimumab	I	17	Objective response was achieved by 3 (17%) patients overall; all 3 had TNBC, so objective response for this group was 43% (n=7).	Metastatic HER2-negative breast cancer	NCT02536794

### 3.7 The role of neoadjuvant immunotherapy in breast cancer

The application of immunotherapy in the neo-adjuvant setting, prior to any operative or adjuvant therapy, is expected to induce more beneficial results for cancer patients. This is based on preclinical evidences in animals that show improved immune responses and better survival rates when immunotherapy was administered before tumor resection or while the primary tumor plus the local lymph nodes were intact (62). This superior response is partly attributed to fact that immunotherapy, in a neoadjuvant environment, primes a stronger anti-tumor immune response prior to the changes in the tumor microenvironment or increased tumor antigen heterogeneity. Considering the attractive anticancer impacts of immunotherapy in treating patients with early-stage disease in the adjuvant setting, research now seeks to utilize ICI blockade for treating such patients in the pre-operative or neoadjuvant setup. A 2019 randomized phase II study, enrolling 174 patients with operable TNBC, administered durvalumab in addition to anthracycline/taxane-based chemotherapy in a neoadjuvant setting- the durvalumab-treated arm demonstrated a superior pCR, particularly in the PD-L1-positive subgroup (63). In agreement, a phase Ib study involving 60 high-risk, early-stage TNBC patients displayed a pCR rate of 60% following a combination of pembrolizumab and chemotherapy as neoadjuvant therapy (64). Interestingly, the results from the study also reported a positive correlation between pCR and PD-L1 expression along with stromal TILs. In addition, the IMpassion 031 study recruiting 333 patients explored the outcome of atezolizumab in conjunction with chemotherapy as neoadjuvant therapy in early TNBC subjects (65). Their results suggested that the combination treatment led to a dramatic increase in pCR rate, implying the potential application of atezolizumab as an alternative therapy for patients with TNBC. Besides, an ongoing MIRINAE study is comparing the efficacy of atezolizumab plus capecitabine versus capecitabine alone among TNBC patients having residual tumors following neoadjuvant therapy (NCT03756298). On a similar note, a phase III study examining pembrolizumab plus chemotherapy as neoadjuvant treatment for early-stage TNBC revealed that pembrolizumab successfully increased the pCR rate. Also, data hinted that patients with a heavier tumor burden, advanced stage of the disease and with positive lymph nodes may especially benefit from pembrolizumab (66). Also, neoadjuvant chemotherapy plus pembrolizumab in 250 patients prior to surgery showed a significantly higher pCR rate among the TNBC population, a result that is consistent with the findings from the KEYNOTE-522 study (32). The I-SPY 2 trial (NCT01042379), involving early-stage TNBC patients, initially showed that pembrolizumab administered with neoadjuvant paclitaxel followed by chemotherapy (doxorubicin and cyclophosphamide) resulted in a notably enhanced pCR rate from 22% to 60% and, this was most probably due to the known immunostimulatory effects of anthracyclines. The efficacy of a treatment regimen constituted of pembrolizumab in combination with paclitaxel plus carboplatin followed by anthracycline plus cyclophosphamide as neoadjuvant therapy prior to surgery, and cycles of pembrolizumab administration as adjuvant therapy, was investigated in the KEYNOTE-522 trial (NCT03036488). The pCR rates escalated from 51.2% to 64.8% (NCT03036488). Extensive follow-up research and long-term immune-related adverse effects need to be thoroughly determined to strengthen the observations. Two ongoing key trials are addressing the effect of a year-long adjuvant anti-PD-1/PD-L1 therapy on the survival of TNBC patients- firstly, the SWOG S1418/BR006 trial (NCT02954874) involving pembrolizumab for patients with residual disease and, secondly, the A-brave trial (NCT02926196) examining avelumab for individuals with high-risk or residual disease. In accordance, two additional trials are inspecting the efficacy of atezolizumab in combination with both neoadjuvant and adjuvant therapy on patient survival outcomes- the placebo-controlled NSABP B-59 trial (NCT03281954) testing the efficacy of atezolizumab plus neoadjuvant chemotherapy prior to adjuvant atezolizumab for one year, and the IMpassion030 trial (NCT03498716) studying the standard adjuvant chemotherapy with or without atezolizumab before an annual regime of adjuvant atezolizumab.

## 4 Emerging concepts to further improve immunotherapy-involving cancer stem cells, tumor infiltrating lymphocytes and microbiota

Extensive research and clinical trials have enabled the slow but gradual integration of immunotherapy as a mainstream treatment strategy in conjunction to existing modules for breast cancer. In addition to the well-established regimes of immunotherapy, as discussed in earlier sections, there is increasing interest in the therapeutic efficacy of other components of the immune system, such as the TILs, as well as various oncogenic modifiers, including cancer stem cells and the microbiota.

### 4.1 Breast cancer stem cells as candidate for immunotherapy

Cancer stem cells, in contrast to other cancer cells, are slow-dividing with a repressed tendency to undergo apoptosis and more agile in terms of DNA repair. These features render the cancer stem cells exceptionally refractory to traditional methods of treatment, like irradiation or chemotherapy. Cancer stem cells are known to express ABC drug transporters, which may explain the underlying mechanism towards their resistant nature (67). Disease relapse and tumor metastasis commonly arise from cancer stem cells that are not affected by traditional anticancer therapy. Elimination of breast cancer stem cells (BCSCs) may be accomplished through immunotherapy, which is likely to improve treatment outcomes for breast cancer patients. Although numerous attempts have been made to target specific CSC markers using preclinical models employing various immunotherapeutic approaches, the biggest hurdle has been posed by the non-uniqueness of these markers since most of them are also expressed by normal stem cells. CSCs found in TNBC patients are highly heterogeneous and dynamic, demonstrating variable responses to chemotherapy. Again, HER2-positive BCSCs are characterized by CD44^high^/CD24^low^ phenotype and ALDH1 expression and they support resistance to anti-HER2 therapy, including trastuzumab. Importantly, this population of cells are frequently detected in recurrent breast cancer and not in primary tumors (68). Immunotherapeutic interventions seek to target BCSCs by utilizing immune cells such as NK cells, CD8^+^ T cells and γδ T cells (69). Till date, many surface markers have been reported for BCSCs including CD90, CD49, CD44, CD24, ALDH and EpCAM (70). Elimination of CSCs was achieved *in vitro* in breast cancer cell lines with ALDH-specific CD8^+^ T cells, which resulted in significant amelioration of mammary tumor development and metastases with prolonged survival (71, 72). Clinical trials are presently investigating CAR-T cells targeting CD44v6 (NCT04430595) and EpCAM (NCT02915445) surface antigens as an effective anticancer module for advanced breast carcinoma. Other studies recorded that BCSC-DCs can effectively inhibit BCSC proliferation when administered into the circulation of BCSC tumor-harboring rodents, suggesting the therapeutic potential of BCSC antigen-primed DCs for elimination of BCSCs (73). These results were further strengthened in additional murine models, wherein BCSC-primed DCs had a positive effect on the survival time by 70% (74).

Vaccination strategies based on DCs encompass either antigen-defined vaccines or polyvalent vaccines (75). In a mouse model of spontaneous mammary tumorigenesis, a DC-based vaccine specifically targeting HER-2/neu led to the production of anti-neu antibodies along with T-cell mediated expression of interferon-γ, resulting in tumor regression (76). Encouraging results were also observed in patients with metastatic breast carcinoma, who were administered with lysate-pulsed DCs (NCT02063893). Nonetheless, immunotherapy approaches that target a single antigen often fail to eradicate the population of cells that contribute to tumor initiation or cancer metastasis; therefore, this significantly lowers the long-term success of this strategy. To circumvent this concern, emerging studies suggest targeting of multiple antigens for an effective response and one way to accomplish this is through polyvalent vaccines. In stage IV melanoma, a DC/irradiated tumor vaccine displayed complete tumor remission in 3 patients and a partial disease remission in an additional 3 out of 46 patients (77). In an interesting study, human heterokaryons were prepared that expressed both breast tumor-associated antigens and costimulatory molecules derived from DCs. These functionally active fusion cells could successfully induce autologous T cell proliferation and stimulate cytotoxic-T lymphocyte activity to fight against autologous breast cancer cells (78). Development of a polyvalent vaccine for BCSCs requires identification of as many antigens as possible that are unique to BCSCs. Determining the presence of mutations that facilitate the stem cell-like phenotype in BCSCs and the underlying mechanisms may unearth important avenues for immunotherapy. Moreover, chemokine receptors that promote migration of BCSCs can also be explored as future targets for immunotherapy. Overall, harnessing DC-based vaccines may be a viable option for targeted elimination of BCSCs (79). Immuno-targeting of BCSCs holds great clinical significance in an adjuvant setting as it can abrogate the BCSC population and can, therefore, improve the outcome of existing therapies.

### 4.2 The involvement of TILs in immunotherapy response in breast cancer

TILs are vital indicators of tumor immunogenicity (80), hence, the presence of TILs serves as a prognostic marker in many malignancies, including breast cancer (81). TILs collectively constitute of the T lymphocytes (CD8^+^, CD4^+^ and T_reg_ cells), B lymphocytes and natural killer cells present within the tumor. These lymphocytes impart crucial functions in breast carcinogenesis and immune recognition. The basic mode of action of CD8^+^ T cells is the induction of direct cytotoxicity to the cancer cells whereas CD4^+^ T cells primarily promote release of inflammatory cytokines to evoke anticancer immunity (82). On the other hand, the CD4^+^ T_reg_ population promotes an immune-suppressive environment by restricting the activation and subsequent function of CD8^+^ T cells (83). TILs are considered responsible for superior disease outcomes among breast cancer patients and are associated with relapse-free survival (84). However, we still do not entirely understand the T cell subtypes in breast carcinoma. One subset of the CD8^+^ TILs is represented by the CD8^+^ tissue-resident memory (TRM) cells that express cytotoxic molecules and immune checkpoint factors (85). These cells were found to positively correlate with higher relapse-free survival in TNBC patients (86). The presence of TRMs also favor improved prognosis among early-stage TNBC patients, denoted by better survival and reduced rates of tumor recurrence. Again, the presence of CD39^+^PD-1^+^CD8^+^ T cells in the tumors is intimately connected with prolonged DFS of breast cancer patients (87). Importantly, the FOXP3^+^ T_reg_ cells contribute to more aggressive outcomes in breast cancer, characterized by an enhanced likelihood of relapse and poor survival (88). A study found that the intra-tumoral infiltration of CD8^+^ T cells led to a notable drop in the risk of death among 12,439 breast cancer patients. This was especially evident for TNBC and HER2+ tumors, who demonstrated a 28% reduction in mortality while ER+, HER2+ tumors had a 27% reduction in mortality (89). TIL therapy involves isolation of TILs from patients and expanding them in an *ex vivo* setup with considerable amounts of IL-2 and other necessary cytokines, followed by their re-infusion into the patient (90). Since TNBC patients express increased number of neoantigens relative to other subtypes, as revealed by whole genome sequencing of breast cancer tissues, TNBC patients may serve as possible candidates for TIL therapy (91). In accordance, preliminary data from an ongoing trial (NCT01174121) has reported tumor regression in a subset of patients in response to TIL therapy (92).

Despite the emerging studies, the effects of the intra-tumoral population of immune cells in dictating response of breast cancer patients to different modes of treatment, specifically immunotherapy, are not fully defined. Importantly, the proportion of the intra-tumoral immune infiltrates is an important factor that determines breast cancer patient response to therapy. In the SweBCG91RT trial, early-stage breast cancer patients possessing immune infiltrates with anti-tumor effects exhibited a lower risk of tumor recurrence (93). Limited benefits were observed in the test subjects following addition of radiotherapy. A high TIL count has been shown to promote sensitization of tumors to chemotherapy, resulting in a high pCR to pre-operative chemotherapy among primary breast cancer patients (94). Another study involving around 3,000 breast cancer patients found that increased TIL counts exerted a survival benefit with an improved response to neoadjuvant chemotherapy in TNBC and HER2-enriched mammary tumors (95). On the contrary, a high TIL count was associated with adverse prognosis in luminal breast cancer. Furthermore, DFS was sharply worse for TNBC patients with TIL^low^ tumors compared to patients with TIL^high^ tumors (96). TILs are, therefore, intimately involved in tumor prognosis, chemotherapeutic outcome and selection of immunotherapy or adoptive cell therapy in TNBC patients (97). Till date, most studies have focused on the prognostic relevance of TILs in breast cancer. However, attractive properties such as diversity of the receptors, tumor specificity, and lack of toxicity have pushed TILs as a promising candidate for therapy (98).

### 4.3 A peek into the role of microbiota as a potent regulator of breast cancer development and response to immunotherapy

#### 4.3.1 Microbiota in breast TME

Distinct differences in the composition of microbiota in the mammary tumor microenvironment of breast cancer patients compared to healthy subjects and, also, between tumor versus adjacent normal tissues have been observed (99). For instance, the abundance of Enterobacteriaceae, Staphylococcus, and Bacillus within the mammary tumor tissues among 71 breast cancer patients was noted (100). Another study found a significant enrichment of *Sphingomonas yanoikuyae* in normal tissues while *Methylobacterium radiotolerans* was abundant in the paired breast tumor tissues (101). *Sphingomonas* is known to regulate estrogen metabolism and activation of pathways associated with Toll-like receptor (TLR) 5, which can affect initiation of breast cancer (102) while colonization by *Methylobacterium* may be involved in ER modulation (101). In general, members of the phyla Proteobacteria, Firmicutes, and Actinobacteria were found to be particularly enriched in breast cancer. Other studies demonstrated reduced presence of the families, Alcaligenaceae, Clostridia, Pseudomonadaceae, Ruminococcaceae, and Sphingomonadaceae, in tumor-adjacent normal tissues relative to mammary tumor tissues whereas Caulobacteraceae, Methylobacteriaceae, Micrococcaceae, Nocardioidaceae, Propionicimonas, and Rhodobacteraceae were enriched in the breast carcinomas (103). The same study reported a decrease in the family Bacteroidaceae with an augmented presence of the genus Agrococcus with advancement of the disease, indicating that the microbiota dynamically changes with breast cancer progression. Furthermore, enrichment of several genera, such as, Fusobacterium, Atopobium, Gluconacetobacter, Hydrogenophaga, and Lactobacillus has been correlated with breast malignancy (99). Decreased breast cancer cell survival due to the presence of *Pseudomonas aeruginosa*, a pathogen found inside the breast was also observed (104). In addition, a study involving 668 breast tumor tissues from The Cancer Genome Atlas (TCGA) data set suggested a strong correlation between EMT-related genes with the presence of *Listeria fleischmannii*, while *Haemophilus influenzae* was associated with tumor growth, cell cycle progression, and mitotic spindle assembly (105). Again, *Staphylococcus epidermidis* facilitate a highly inflammatory tumor microenvironment, through induction of pro-inflammatory cytokines and complement activation, which favored tumor growth while treatment with antibiotic ameliorated these effects (106). Fu et al. further showed the presence of tumor-resident microbiota in a spontaneous murine breast cancer model that stimulated metastatic progression (107). However, the precise effects of these breast tumor-residing microbes on response to immunotherapy remain to be investigated and validated.

#### 4.3.2 Pleiotropic effects of microbial metabolites on breast carcinogenesis

The intestinal microbiota is responsible for the production of short-chain fatty acids (SCFAs), such as acetate, butyrate, lactate, and propionate, which are important constituents of the tumor microenvironment. Microbial metabolites enter the circulation and exert pleiotropic anticancer effects in target cells. Interestingly, the presence of SCFA-producing bacteria was found to be considerably decreased among premenopausal patients with breast cancer in comparison to healthy premenopausal women (108). Microbial dysbiosis alters the bacterial metabolites to favor multiple hallmarks of cancer, including cell proliferation, apoptosis, metabolism, invasion, inflammation and immune regulation (109, 110). Sodium butyrate, for example, enhances oxygen consumption in breast cancer cells (111). Increased breast cancer cell death is observed following treatment with butyrate or inhibition of lactate metabolism (112, 113). SCFAs reportedly crosstalk with the immune environment as well, and are known to stimulate secretion of cytokines, such as IL-17, IFN-γ, IL-10 among others, and promote T cell differentiation. Butyrate has been shown to metabolically rewire activated CD8^+^ T cells that influences the transition of CD8^+^ T cells to memory cells (114). A recent study in a cohort of TNBC patients demonstrated a correlation between enrichment of Clostridiales in tumor tissues with an activated immune microenvironment. This bacterium is responsible for the production of metabolite, trimethylamine N-oxide, which imparts activation of M1 macrophages and CD8^+^ T cell-mediated antitumor response, thus opening avenues for understanding its effect on immunotherapy (115). In melanoma patients, responders to immunotherapy usually exhibit an abundance of butyrate-producing microbiota relative to non-responders (116). Additionally, butyrate improved the efficacy of anti-PD-1 therapy *via* enhanced T cell infiltration in the tumor microenvironment in colorectal carcinoma murine model (117). Although such evidence clearly point towards the close interactions between microbial metabolites and the immune system, there is a lack of understanding regarding the mechanisms of metabolite-induced changes in immunotherapeutic response across breast cancer patients. Future studies should also focus on the implications of supplementation with such metabolites as an adjunct regime for immunotherapy in breast cancer.

#### 4.3.3 Modulation of the microbiota as a strategy for overcoming resistance to immunotherapy

**Figure 2:**
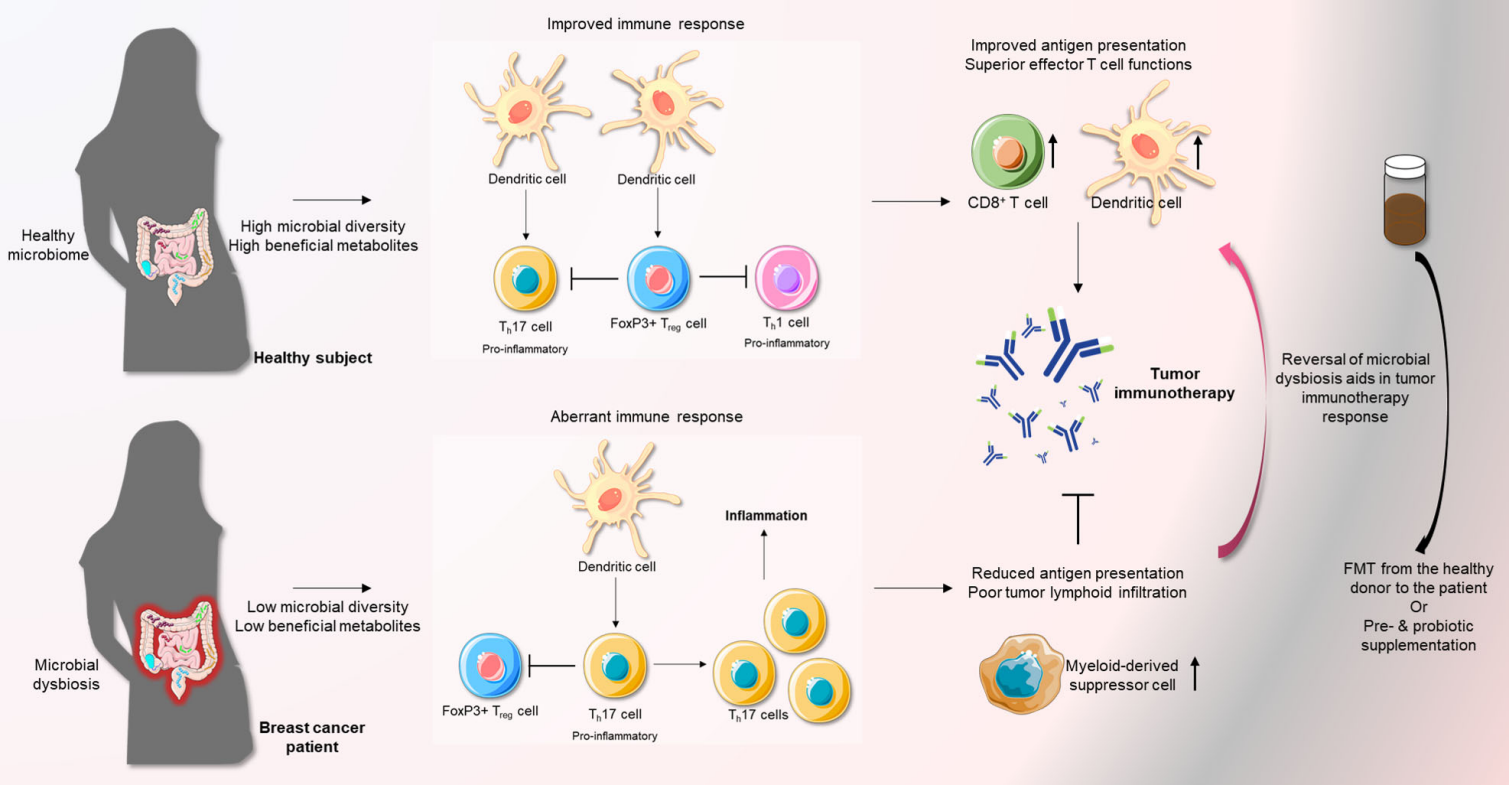
A favorable microbiota strengthens the anti-tumor immune response mediated by immunotherapy. Normally, a healthy microbiota maintains a state of equilibrium of T_h_17 cells and FoxP3+ T_reg_ cells. Overall, this inhibits pro-inflammatory immune responses. However, microbial dysbiosis, an important risk factor of breast cancer, can abrogate this regulation and induce a state of inflammation by favoring T_h_17 pro-inflammatory T cells over regulatory FoxP3+ T cells. This can severely impede the effects of immune-therapy. Remodeling of the microbiota in breast cancer patients through the use of FMT or supplementation with pre-/probiotics can facilitate a more favorable state of immune response, especially in the presence of immunotherapy.

Multiple studies have confirmed the involvement of host microbiota in oncogenesis and therapeutic response (118). A seminal study showed the attenuated effects of anticancer treatment in mice with depleted gut microbiota (either due to treatment with an antibiotic or housing in germ-free conditions), suggesting that the host microbiota is a critical determinant of therapeutic response (119–121). Study examining the effects of gut microbiota on tumor suppressive efficacy of trastuzumab in HER2-positive breast cancer revealed that antibiotic exposure or FMT from antibiotic-treated mice greatly impair the antitumor activity of trastuzumab (122). In fact, HER2-positive breast carcinoma patients refractory to trastuzumab treatment, demonstrated a lower α-diversity and reduced abundance of *Bifidobacteriaceae, Prevotellaceae*, *Lachnospiraceae*, and *Turicibacteraceae* compared to individuals who achieved pCR (122). A direct interaction between the gut microbiota and patient responsiveness to therapy implies that modulation of the gut microbiota may be explored to achieve optimal ICI efficacy. As microbial dysbiosis strongly influences local and systemic antitumor immune response (119), an intricate connection between ICIs’ efficacy and host microbiota has also been observed. Gut microbial community strongly influences the antitumor immune responses through modulation of CD8^+^ T cells, T helper 1 (T_h_1) and tumor-associated myeloid cells (120, 121, 123). Multiple landmark efforts, subsequently, in murine models recognized the association between the gut microbiota and ICI effectiveness. Responses to anti-PD-L1 therapy alter based on the gut microbiota composition which can be modulated with fecal microbial transfer (FMT) or co-housing approach. Of note, oral administration of *Bifidobacterium* augmented the maturation of DCs and CD8^+^ T cells priming and tumor infiltration, which restored the antitumor efficacy of PD-L1 therapy (124). In agreement, supplementation with *Bacteroides fragilis* along with *Bacteroides thetaiotaomicron* or *Burkholderia cepacian* enhanced the anti-tumor effects of anti-CTLA-4 blockade in microbiota-depleted mice (125). Other studies also showed enhanced efficacy of ICIs *in vivo* following treatment with several bacterial strains such as *Lactobacillus johnsonii*, *Bifidobacterium pseudolongum*, and *Olsenella* species (126). Interventions such as FMT, probiotic and prebiotic supplementation are presently being interrogated to determine the impact of restoration of the gut microbiota on therapeutic efficacy of various modes of immunotherapy (**Figure 2**). For instance, a clinical trial in patients with breast cancer is delineating the outcome of probiotics administration (13 strains of beneficial bacteria) on CD8^+^ T cell infiltration in the tumor microenvironment (NCT03358511). The collated evidence, thus, points to the need for future clinical research to test if manipulation of the host microbiota may aid in improving immunotherapy outcomes in patients with breast carcinoma.

## 5 Perspectives of combined therapy modules in breast cancer and avenues for future research

Currently, despite its immense potential, the efficacy of immunotherapy as monotherapy is quite limited in solid tumors. Emerging results clearly point towards the benefits of the combinatorial approaches involving immunotherapy and conventional treatment modules but there are certain aspects that demand additional in-depth research, such as the precise timing of intervention, optimal drug combinations, and the order of administration of drug combinations. Identifying potentially responsive tumors is also extremely important as the efficacy of ICIs varies among all tumor types and, in certain cases, there is the occurrence of immune-related adverse events (irAEs). In addition to PD-1/PD-L1 inhibitors, other immunotherapy modalities, such as CTLA-4 inhibitors, CAR T cell therapy and tumor vaccines are also being investigated in combination strategies. Development of vaccines to enhance anticancer immunity is another upcoming strategy to target breast cancer. Presentation of breast cancer peptides to T cells through these vaccines can stimulate T cell priming and activation in addition to boosting immune recognition of cancer cells. At present, several clinical trials with a goal to identify the efficacy of breast cancer vaccines in combination with PD-1/PD-L1 inhibitors in TNBC setting are ongoing. Interestingly, neoantigen vaccines are designed to target the peptides procured from tumor-specific mutations, absent in normal cells, and unique to the tumor of the patient for minimizing self-tolerance (127). A randomized phase I study will determine the impact of a neoantigen vaccine plus durvalumab among patients with residual TNBC following neoadjuvant therapy (NCT03199040). Another phase II trial is enrolling mTNBC patients, who have not been exposed to any form of treatment, in addition to those mTNBC subjects, who have been treated with chemotherapy (gemcitabine and carboplatin) for 18 weeks, to examine the effects of nab-paclitaxel plus durvalumab in conjunction with a neoantigen vaccine (NCT03606967). Another advance in immunotherapy repertoire is the CAR T cell therapy engineered for specific targeting of tumor antigens. Albeit preliminary, studies have determined that intra-tumoral administration of engineered CAR T cells does not elicit any serious adverse effects in patients with metastatic breast cancer (NCT01837602) (128). These upcoming promising immunotherapies warrant additional preclinical, translational and clinical studies to improve the existing treatment regime for breast cancer patients.

Results from the current trials suggest that TNBC patients at earlier stages of the disease responded better to combination therapy but the prognosis of advanced TNBC has scopes for considerable improvement. More elaborate studies need to be designed for assessing the long-term synergistic interactions between immunotherapies with chemotherapies. Efforts are required to consider the plausible toxicity profile that may be associated with such new treatment modalities. Since the immune system is highly variable from person to person, studies need to focus on the differential tolerance to such combination therapies amongst different cohorts of patients. Customization of precision immunotherapies assisted through predictive biomarkers is expected to enhance the clinical efficacy and responsiveness to therapy among patients, thus making this an important and interesting area of further research. Emerging studies have pointed that race may be a contributing factor to dictating the responsiveness of breast cancer patients to therapy- a recent study showed the role of racial disparity in response to immunotherapy among Asian breast cancer patients (129). This underscores the importance of conducting investigations to ensure the effectiveness of the combination approaches for immunotherapy among breast cancer patients. For TNBC patients, we need to explore predictive markers to identify the responders versus non-responders across TNBC subtypes, such as basal-like, mesenchymal stem cell-like, etc. which promotes the observed heterogeneity in clinical efficacy of the combinatorial immunotherapy-based strategies. Despite the promising potential of the combination strategies for breast cancer patients, the extremely high cost of this type of treatment makes it relatively hard to pursue, especially for a long duration. Consequently, future studies should try to implement better ways to make this form of therapy reasonably feasible and accessible for all compliant patients.

Clinical and preclinical data indicate the presence of complex and dynamic interactions between various components of the immune system that need to be further comprehended to achieve improved treatment outcomes. Although immune-based treatment modalities have gained momentum in the last few years as key therapy in multiple cancers, more rigorous clinical trials are required to prove the clinical efficacy of these agents in breast carcinoma. Modulation of the tumor microenvironment represents an unexplored area of increasing interest as this can be altered to facilitate drug delivery and improve cytotoxicity. For instance, antiangiogenic therapy has not yielded significant results for the treatment of HER2-positive breast cancer patients (130), but immune evasion through CD8^+^ T cell suppression or other mechanisms brought upon by proangiogenic stimuli, such as increased VEGF production, supports the idea of developing antiangiogenic agents in conjunction with ICI as a novel therapeutic approach (131). The innovation of immunotherapies to target HER2-positive breast carcinoma requires close attention to the concerns of favorable efficacy to toxicity ratio. Notably, contemporary evidences suggest that HER2-directed vaccines exhibit favorable toxicity profiles with minor side-effects while adoptive T cell-based therapies have, unfortunately, been associated with greater side-effects (132). Multiple small studies established that a decrease in TIL counts and PD-L1 expression is mostly more common in metastatic lesions relative to primary breast tumors (133, 134). In agreement, one study with paired primary and metastatic breast cancer samples unveiled that metastatic breast cancer tissues were characterized by the downregulation of immunotherapy drug targets, pro-inflammatory cytokines and antigen presentation, along with upregulation of molecules that support immunosuppression (135). Such results hint at the immune-depleted nature of metastatic breast cancers compared to primary tumors. Therefore, a combinatorial approach to enhance the immune response of metastatic breast cancer may prove more beneficial for such immunologically inert tumors. In addition, a more thorough and intensive understanding of the tumor microenvironment may successfully enable a durable and potent anti-tumor response from the combination therapies. Despite these hurdles, activation of the immune system is closely related to self-sustaining and prolonged tumor suppressive actions and numerous patients are likely to benefit from well-designed immunotherapies with limited side-effects.
